# Competency-based veterinary education: an integrative approach to learning and assessment in the clinical workplace

**DOI:** 10.1007/s40037-015-0172-1

**Published:** 2015-03-27

**Authors:** Harold G. J. Bok

**Affiliations:** Faculty of Veterinary Medicine, Quality improvement in Veterinary Education, Utrecht University, Yalelaan 1, 3584 CL Utrecht, The Netherlands

**Keywords:** Workplace-based assessment, Programmatic assessment, Feedback, Competency-based learning

## Abstract

When graduating from veterinary school, veterinary professionals must be ready to enter the complex veterinary profession. Therefore, one of the major responsibilities of any veterinary school is to develop training programmes that support students’ competency development on the trajectory from novice student to veterinary professional. The integration of learning and assessment in the clinical workplace to foster this competency development in undergraduate veterinary education was the central topic of this thesis.

## Introduction

Veterinary professionals are increasingly playing an important role in the relationship between animals, their health and well-being, and public health [1, 2]. This approach to animal, human and environmental health emphasizes the importance of non-technical competencies in addition to specific technical competencies [3–5]. To develop training programmes that prepare students for entering the complex veterinary profession, competency-based learning and assessment strategies could be a valuable approach. For competency-based education to be successful, content, educational strategies, teaching methods and assessment need to be aligned in a framework of clearly stated programme outcomes. For this purpose, medical curricula tend to use frameworks that define competency domains or roles as areas specific to the profession [6, 7].

In the veterinary clinical workplace, students interact with patients, clients, specialists, and other health care workers. This provides an environment in which learning and assessment can be integrated and focused on the exchange of performance-relevant information [8] in order to enhance competency development [9]. Such an approach to education requires assessment methods and strategies that provide meaningful feedback and stimulate students’ active participation, and adequately assess competency development. The aim of this thesis was to study how an integrative approach to learning and assessment may be able to foster competency development in the clinical workplace. A review of the pillars on which such a curriculum in undergraduate veterinary education should be founded resulted in the research questions posed in this thesis. First, competency-based veterinary education requires educational strategies that are aligned and based on an agreed competency framework. An integrative veterinary competency framework that reflects the scope of today’s veterinary professional practice can serve to guide the development of educational programmes along the continuum from novice student to practising veterinarian. In veterinary literature there was no clearly defined integrative approach to curriculum development that is underpinned by a framework of competencies. This led to the first research question:

- What overarching competency structure provides a foundation for competency-based education in veterinary medicine?

Second, recent developments regarding the interface between learning and assessment have led to a theoretical model that may support an integrative programmatic approach to learning and assessment [10]. However, it was still unclear how this theoretical model interacts with practice when it is implemented in an undergraduate competency-based curriculum. This therefore raised the second research question, which was:

- How does theory interact with practice when implementing a competency-based assessment programme in undergraduate veterinary education?

Finally, the interaction between students and teachers was pivotal in creating information that helps students develop their competencies in the clinical workplace. Further research was required in order to shed light on the underlying mechanisms that affect the exchange of performance-relevant information in competency-based workplace learning and assessment. The final research question addressed in this thesis was:

- Which underlying mechanisms affect the exchange of performance-relevant information in competency-based workplace learning and assessment?

## Methods

The research described in this thesis was based on a design-based research (DBR) approach to contribute towards theory testing and refinement on one hand and enhance educational practice on the other hand [11]. Design-based education research explores the interaction between theory and practice and takes place in authentic learning environments. DBR typically moves forward in cycles of design, evaluation, and redesign [11, 12]. The six studies comprising this thesis described one cycle of design and evaluation, and mainly used qualitative research methods as they generate rich data that can lead to deeper understanding of differing perspectives. This approach allowed us to develop a competency framework for the veterinary professional and to explore how and why complex phenomena in the veterinary clinical workplace occur [11].

Our first two studies looked at which overarching structure of relevant competencies for the veterinary profession could provide a foundation for competency-based education in veterinary medicine. In our first study, aiming to define a competency framework for veterinary professionals, we conducted focus group interviews and a Delphi procedure with participants representing the full range and diversity of the veterinary profession. In the second study we explored international perspectives towards the competency framework that resulted from study 1. A questionnaire was used with veterinarians in 10 countries to explore the degree of international consensus on what could be expected from a veterinary professional, and what should be taught in veterinary education. In our third study we investigated the development, implementation and evaluation of a competency-based assessment programme informed by current theories on programmatic assessment to fit the competency framework designed and validated in studies 1 and 2. Group interviews were used that explored students’ and teachers’ experiences with the competency-based assessment programme. In study 4, 5 and 6 we took a closer look at which underlying mechanisms affect the exchange of performance-relevant information in competency-based workplace learning and assessment. In study 4 we used semi-structured individual interviews to explore students’ feedback-seeking behaviour in the clinical workplace. During these interviews students were asked about their goals and motives for seeking feedback, the characteristics of their feedback-seeking behaviour and factors influencing that behaviour. The teacher’s perspective on interaction between teacher and student in the clinical workplace was explored in study 5. We focused on investigating teachers’ use of the Mini Clinical Evaluation Exercise in performance evaluations to provide narrative feedback. Finally, in study 6, we discussed a social cognitive model of motivation (implicit self-theories) [13] to help explain different kinds of behaviour that emerge when individuals are confronted with challenges, in this case seeking and providing constructive feedback in the veterinary clinical workplace. Current literature on self-theories [14, 15] was used to explore the relevance of these theories in relation to teachers’ and students’ behaviour.

## Results

1. Studies 1 and 2 resulted in an integrated competency framework for veterinary professionals—VetPro—which could provide a foundation for competency-based education in veterinary medicine. The framework contains 16 competencies organized around seven domains: Veterinary Expertise, Communication, Collaboration, Entrepreneurship, Health and Welfare, Scholarship, and Personal Development. The VetPro framework adds to existing competency frameworks in that it is an integrative, holistic approach, placing the Veterinary Professional at the heart of the framework, which focuses on the ability to perform complex professional activities while combining different competencies [16].
2. Study 2 indicated that from an international perspective there is a high degree of consensus amongst veterinarians on what could be expected from a veterinary professional and what should be taught in veterinary education. The VetPro framework proved to be a valid and valuable starting point for opening up an international discourse on the definition of competent veterinary professionals [17].
3. The implementation of an integrative competency-based approach to learning and assessment in undergraduate veterinary education demonstrated two main repetitive challenges. First, using formative assessments to maximally enhance students’ competency development by providing high-quality and meaningful feedback was difficult for faculty. Secondly, when including performance-relevant information from formative assessment instruments in high-stakes assessment procedures, students perceived that feedback as summative, as assessment *of* learning, instead of an assessment *for* learning [18].
4. Three main categories of interrelated factors were identified in studies 4 and 5 as main underlying mechanisms of influence on the challenges of providing rich and meaningful feedback and the problems related to the combination of formative and summative assessment. Personal, interpersonal, and contextual factors seemed to be of influence on students’ feedback-seeking and teachers’ feedback-giving behaviour in the veterinary clinical workplace. Depending on the outcome of an assessment between expected negative effects and potential benefits, these factors gave rise to specific behaviour [19, 20]. For example, students’ and teachers’ personal goals and beliefs in relation to competency development and learning influenced their behaviour towards students’ self-directed learning and the seeking and providing of narrative feedback. This included the extent to which an individual believes that certain attributes are malleable, and the context-dependent goals that an individual sets for him or herself. For example, students who were eager to master a specific clinical task were likely to let expected benefits from seeking feedback prevail over expected costs. In addition, the quality of the relationship between student and teacher and the extent to which a supportive learning culture was created (i.e. a clinical environment that included time for assessment), turned out to be mediating factors in the exchange of performance-relevant information [19–21].

## Discussion

The studies reported in this thesis were part of a design-based research approach that aimed to contribute to conceptual refinement and improvement of educational practice in clinical training. We were able to develop an integrative competency framework that could serve as an underlying structure in the educational strategies (teaching, learning and assessment activities), and in the alignment between these strategies in order to optimally support students’ competency development. In the selection of educational strategies the focus should lie on the integration of all competency domains relevant for the veterinary professional. Subsequently, defining which core professional activities are the constituting elements of the profession could provide the opportunity to align the competency domains with veterinary workplace learning.

The exchange of performance-relevant information during workplace learning and assessment is influenced by a variety of personal, interpersonal and contextual factors. For competency-based education in the veterinary clinical workplace to be successful these points need to be addressed. The results of our studies pointed out that a professional learning culture that allows and enhances the provision and seeking of feedback has a positive influence on the integration of learning and assessment in the clinical workplace. These learning cultures in which observations and feedback are incorporated in normal daily practice create opportunities to exchange narrative, meaningful and task-related feedback. Furthermore, it will enhance validity as assessment is aligned with the core professional activities [22]. To conclude, by investing in relationships between students and teachers, for example by implementing longitudinal clerkships with supervisory continuity, the perceived costs of seeking and providing feedback could be reduced, while enhancing the exchange of performance-relevant information in a competency-based programmatic approach to learning and assessment.

## Conclusion

In this thesis, we have developed a competency framework that could serve as a foundation for curriculum development, and initiated an international discussion on the needs of the veterinary professional in the 21st century. By implementing a competency-based programmatic approach to learning and assessment, we have pointed out important challenges that influence the exchange of performance-relevant information. We studied these challenges within the context of veterinary workplace learning from both students’ and teachers’ perspectives, as well as through discussing research from other domains.
